# Plaque Structural Stress: Detection, Determinants and Role in Atherosclerotic Plaque Rupture and Progression

**DOI:** 10.3389/fcvm.2022.875413

**Published:** 2022-07-07

**Authors:** Sophie Z. Gu, Martin R. Bennett

**Affiliations:** Section of Cardiorespiratory Medicine, Department of Medicine, University of Cambridge, Cambridge, United Kingdom

**Keywords:** plaque structural stress, atherosclerosis, plaque rupture, computational modeling, intravascular imaging

## Abstract

Atherosclerosis remains a major cause of death worldwide, with most myocardial infarctions being due to rupture or erosion of coronary plaques. Although several imaging modalities can identify features that confer risk, major adverse cardiovascular event (MACE) rates attributable to each plaque are low, such that additional biomarkers are required to improve risk stratification at plaque and patient level. Coronary arteries are exposed to continual mechanical forces, and plaque rupture occurs when plaque structural stress (PSS) exceeds its mechanical strength. Prospective studies have shown that peak PSS is correlated with acute coronary syndrome (ACS) presentation, plaque rupture, and MACE, and provides additional prognostic information to imaging. In addition, PSS incorporates multiple variables, including plaque architecture, plaque material properties, and haemodynamic data into a defined solution, providing a more detailed overview of higher-risk lesions. We review the methods for calculation and determinants of PSS, imaging modalities used for modeling PSS, and idealized models that explore structural and geometric components that affect PSS. We also discuss current experimental and clinical data linking PSS to the natural history of coronary artery disease, and explore potential for refining treatment options and predicting future events.

## Introduction

Plaque destabilization results from the complex interplay between structural plaque features, local haemodynamic forces, and biological processes acting within and on the plaque surface. Plaque rupture accounts for ~2/3 of myocardial infarctions (MIs) and sudden cardiac deaths, and results from transmural fissuring of the fibrous cap; in contrast, plaque erosion accounts for approximately 30% of sudden cardiac deaths, and is characterized by an intact and thick fibrous cap, but local endothelial cells are missing (1). While the local haemodynamic environment promotes both plaque rupture and erosion (2), rupture appears particularly associated with mechanical strain (3) and specific plaque types. For example, rupture occurs most frequently in thin-cap fibroatheromas (TCFAs), and fibrous cap thickness (FCT) is an important predictor of rupture. Fibrous cap thinning involves the gradual loss of smooth muscle cells, thereby reducing collagen production (4), and accumulating macrophages secrete metalloproteinases that can degrade the extracellular matrix (5). The cap margin or shoulder region are often the weakest areas in eccentric plaques, although rupture also occurs within the central cap. Cap rupture can present with acute coronary syndromes (ACS), but high-grade stenosis is often associated with clinically silent ruptures (6). However, plaque rupture and progression are not solely determined by plaque strength, but also by mechanical factors that impose stress on lesions.

Coronary arteries are under constant mechanical loading, with blood pressure and flow being the predominant externally-applied loads. Coronary arteries also experience internal stresses that depend on both externally-applied loads and residual stress of arterial wall constituents. Plaque structural stress (PSS) and wall shear stress (WSS) are two important forces involved in plaque development and rupture. PSS refers to stress within the plaque as it deforms under physiological arterial pressure, with three principal stresses acting in longitudinal, circumferential and radial directions at every point. Maximum (or peak) principal stress is the highest of these stresses, is usually directed circumferentially, and is ~100–300 kPa. In contrast, WSS refers to tangential stress resulting from friction of blood flowing on the endothelial surface, and is typically ~1Pa (10 dynes/cm^2^) (7). The effect of haemodynamic forces on plaque formation and destabilization was first proposed in 1969 (8), and now a large body of evidence demonstrates that mechanical forces result in biological effects on the vessel wall.

## Definition Of Plaque Structural Stress

**Figure 1:**
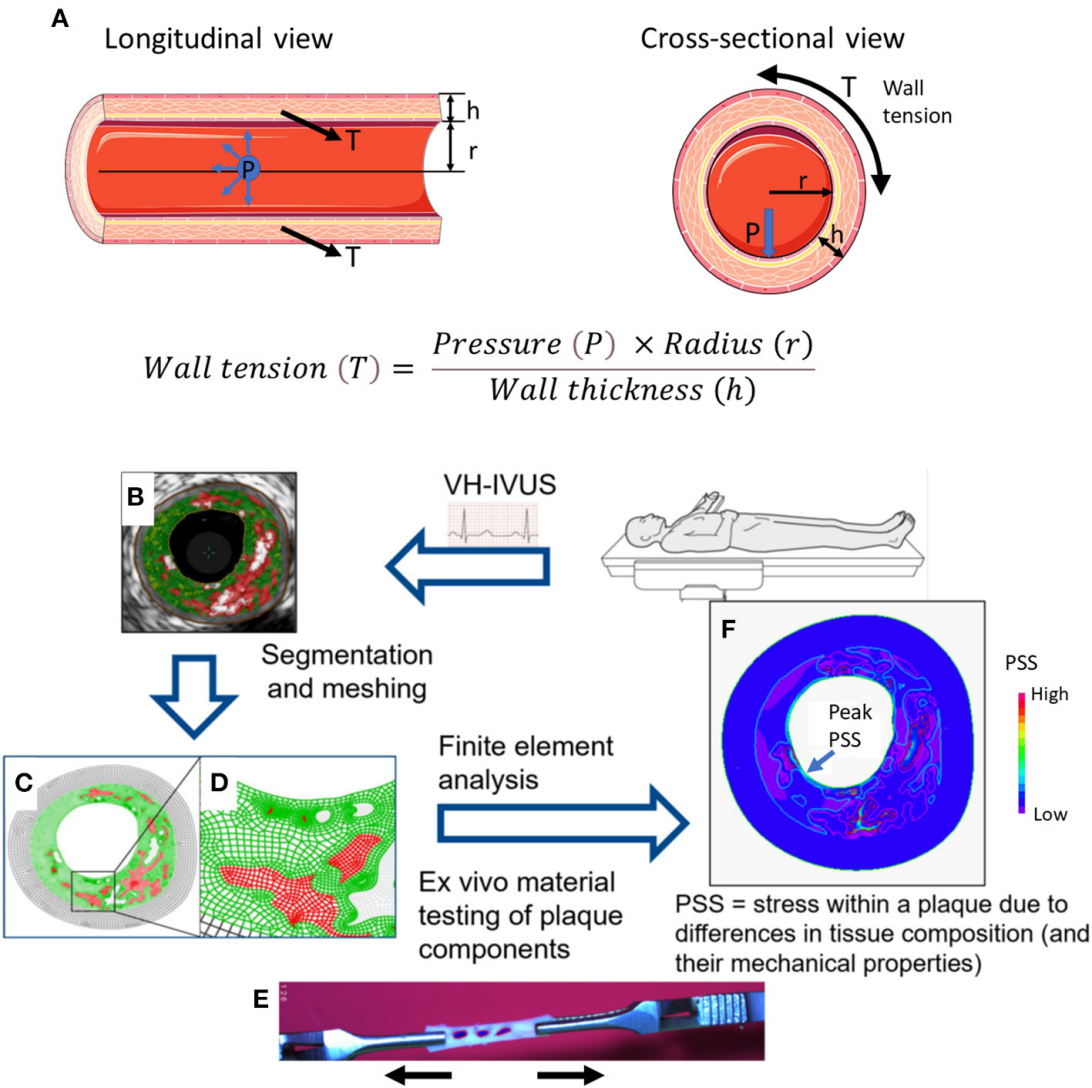
Modeling of plaque structural stress. **(A)** The law of Laplace describes the relationship between transmural pressure (P) and wall tension (T). In a (cylindrical) blood vessel, there is a simple relationship between pressure and circumferential wall tension/stress. The law gives the average tension over the wall, but holds only for simple geometries. h = wall thickness; r = radius. Example of steps involved in computational modeling to calculate plaque structural stress (PSS): **(B)** Suitable images (e.g., virtual histology intravascular ultrasound, VH-IVUS) showing plaque structure and components generated from *in vivo* or *ex vivo* studies; **(C,D)** Images undergo segmentation and meshing process; **(E)** Plaque material properties are obtained from *ex vivo* tensile testing of plaque components; **(F)** Finite element analysis (FEA) utilizes plaque geometry, structure, components, material properties, and haemodynamic conditions to generate a numerical solution of PSS. Adapted from Brown et al. (16).

PSS is the mechanical stress located within an atherosclerotic plaque or the arterial wall, and varies with vessel expansion and stretch induced by arterial pressure and heart motion. PSS is determined by multiple factors, including plaque composition, geometry and blood pressure (7). As hydrostatic and dynamic blood pressure cause arterial expansion, the walls attempt to resist this deformation, resulting in changes in PSS in systole and diastole. For computational modeling, the coronary vessels are considered as thin-walled cylinders with pressures applied to the vessel wall from within. Radial stress is often neglected in thin-walled vessels being small compared to circumferential stress, such that PSS is synonymous to circumferential wall stress (or wall tension, T). Circumferential forces act upon every particle in a cylinder wall, and stress is loaded across the vessel wall tangentially, similar to increasing circumferential wall stress as a balloon expands when inflated. Thus, PSS increases with luminal area/radius, and decreases with luminal stenosis and increasing vessel/plaque thickness, as governed by Laplace's law (Figure 1A):


$$\begin{array}{r} Wall\;tension\;\left(T\right)=\;\frac{Pressure\;\left(P\right)\;\times Radius\;\left(r\right)}{Wall\;thickness\;\left(h\right)} \end{array}$$

## Modeling Plaque Structural Stress

Computational modeling of tissue stresses and strains induced by solid and fluid mechanics uses partial differential equations governed by physical principles. In particular, PSS calculation requires knowledge of mechanical properties of plaque components, magnitude of externally applied loads, and extent of resultant plaque deformation. Material properties govern how a tissue behaves under varying loading conditions and are typically described by the stress-strain relationships. For linear elastic materials that act uniformly in all orientations (isotropic), material behavior is described by Young's modulus (*E*):


$$\begin{array}{r} E=\;\frac{Stress}{Strain} \end{array}$$

and Poisson's ratio, the negative ratio of transverse to axial strain. Material properties for different plaque components are estimated by uni-extension tests, which can be problematic as atherosclerotic tissue is small, fragile and rarely comprises a single component. Biological tissues also behave differently depending on direction of applied force (“anisotropy”), being stiffer in axial and circumferential directions than radial. Nevertheless, these approximations can be used for PSS calculation where vascular wall and plaque components are considered as hyperelastic, and their stress-strain relationship expressed as a strain energy density function (SEDF), most commonly the Mooney-Rivlin and Neo-Hookean constitutive equations (7).

## Computational Modeling By Finite Element Analysis (Fea)

The complexity of atheromatous plaques requires reconstruction of either 2D or 3D arterial solid models from medical imaging, with plaque segmentation into smaller domains comprising individual elements (Figures 1B–D). Computational techniques such as FEA allow stress calculations from a number of variables, including plaque geometry and composition, tissue material properties (from *ex-vivo* tensile testing, Figure 1E) (9, 10) and haemodynamic forces. FEA incorporates all this information into a solution for each element and ultimately the entire area of interest, resulting in PSS estimates and its variation during one cardiac cycle (Figure 1F).

Thus, a standard approach for modeling plaque solid and/or fluid mechanics is:

1. Dynamic loading conditions estimated using invasive (e.g., coronary pressure at invasive coronary angiography) or non-invasive (e.g., blood pressure) methods.
2. Image reconstruction into 2D or 3D vessel and plaque geometry, with suitable computational meshing.
3. Model assumption of flow conditions, boundary conditions, and material properties.
4. Suitable computational methods (e.g., FEA) to solve for plaque stress, using plaque geometry, tissue material properties, and haemodynamic forces.
5. Computation performed on chosen discretised model of SEDF (e.g., Mooney-Rivlin or Neo-Hookean) to describe material properties of each plaque component, and their motion by kinetic equations.
6. Illustration of calculated stresses.

Similar to FEA, computational fluid dynamics (CFD) is based on solving Navier-Stokes equations governing fluid mass and flow to describe fluid motion. Combining CFD and FEA allows simultaneous analysis of solid and fluid domains, termed fluid-structure interaction (FSI).

## Intravascular Imaging Modalities For Biomechanical Modeling

Intravascular ultrasound (IVUS) is a widely-used, invasive, catheter-based tool to assess atherosclerotic burden. Greyscale IVUS (GS-IVUS) provides real-time, 2D cross-sectional, monochrome images of coronary plaques, but cannot define plaque composition or components accurately. In contrast, spectral analysis of radiofrequency ultrasound backscatter data [radiofrequency- or virtual histology-IVUS (IVUS-RF or VH-IVUS)] can provide automatic assessment of four main plaque components, including dense calcium, fibrofatty tissue, fibrous tissue, and necrotic core (11). Although the resolution of VH-IVUS is insufficient to identify thin fibrous caps, the ability to provide both anatomical and compositional information means that IVUS-RF/VH-IVUS is a good imaging modality for PSS calculation by FEA, and suitable for stable, vulnerable or ruptured plaques. For example, while prospective studies found that VH-IVUS can identify plaque features associated with MACE, including plaque burden >70%, minimal lumen area <4 mm^2^, and VH-defined TCFA (12–14), PSS estimations provided incremental prognostic information to these studies (15–17).

Optical coherence tomography (OCT) uses near infrared light to generate ultra-high-resolution images. OCT's spatial resolution of 4–20 μm (18) allows measurement of FCT, and FCT <60 μm, presence of macrophages, and neovessels have been identified as higher-risk features in prospective studies (19–21). OCT can also identify plaque components including fibrous tissue, lipid and calcium (22), potentially useful to estimate PSS. However, poor penetration of near infrared light (<2 mm, depending on tissue type), light signal attenuation, and absorption issues limit visualization to the plaque surface, restricting its ability to define plaque burden. In addition, OCT signals are highly attenuated by large lipid pools, preventing border identification. While new automatic methods are being developed to characterize plaque composition from OCT (23, 24), their accuracy to define deep plaque structures and borders is unclear. OCT also performs poorly in larger lumen arteries, tissues behind the guidewire shadow cannot be visualized, and inability to progress the guidewire can restrict imaging with large plaques or stenosis. As whole plaque structure is required for solid mechanics modeling, PSS calculation using FEA on OCT images is challenging, although (as yet unproven) may be possible in central (carotid) or peripheral (femoral) arteries.

In contrast, hybrid IVUS-OCT imaging combines advantages associated with each technology, including superior resolution of OCT to measure FCT and better penetrance of IVUS to visualize deeper plaque structures and assess arterial remodeling (25). Combined hybrid imaging improves plaque classification in *ex vivo* human coronary and post-mortem studies (26, 27), and integrated IVUS and OCT single catheters have been developed (28). Importantly, combined IVUS-OCT catheters utilize a single pullback and avoid time-consuming and error-prone image co-registration, providing a promising imaging system for solid mechanics plaque stress modeling.

Other intravascular imaging tools such as near-infrared spectroscopy (NIRS) in their current form have limited utility for computational modeling. For example, while NIRS can identify and quantify lipid cores (29) and lipid core burden index (LCBI) or the 4 mm segment with maximum LCBI can identify higher risk plaques (30, 31), NIRS cannot evaluate depth and volume of lipid cores or identify other plaque components accurately. Similarly, the limited resolution of non-invasive imaging [0.5 mm for computed tomography, 1–1.5 mm for magnetic resonance angiography (32)] makes it challenging to identify coronary plaque structure accurately, although reasonable vessel structure for solid biomechanical modeling can be obtained in larger arteries (33–35).

## Pss In *Ex-Vivo* And Idealized Studies Of Atherosclerotic Plaques

Both *ex vivo* and *in vivo* studies implicate high PSS in plaque rupture. For example, FEA of *ex vivo* histology sections of human arteries demonstrates that ruptured plaques are associated with increased PSS. In addition, reduced FCT, increased necrotic core, and microcalcification are all associated with higher-risk plaques, and decreasing FCT, increasing necrotic core area and microcalcification significantly elevate PSS in idealized 2D FEA models (36–38). Increased necrotic core thickness also results in higher circumferential stress in experimental models, whereas increased FCT has the opposite effect. In contrast, increased vessel stenosis (decreased lumen diameter) reduces circumferential stress (in accordance to Laplace's law), so it cannot be assumed that PSS increases as lesions progress.

Idealized 3D models confirm these observations, demonstrating that smaller lipid pools reduce maximum stress, while PSS increases by 30% when FCT is halved (39), and can exceed 300 kPa when FCT is <60 μm (a marker of higher-risk plaques), irrespective of plaque geometry (40). Luminal curvature also significantly affects location of peak PSS (41), with peak values typically occurring at plaque shoulders, although can also occur in the fibrous cap center. Another key determinant of PSS is the size, orientation, shape, and connectivity of individual calcium deposits, with high stress areas located at interfaces between calcified and non-calcified tissue (7). For example, deep calcification within the plaque has little overall effect on surface PSS, while superficial calcification adjacent to lipid core can attenuate PSS (42). In addition, FEA models predict that larger plates of calcification (generally >1 mm in size) reduce PSS, whereas small foci of calcium, termed microcalcification, significantly increase PSS (38). Interestingly, maximal PSS values within plaques are often not located on the calcium itself, but instead just upstream of the deposit (43). Microscopic, cellular-level microcalcifications (~10 μm diameter) accumulate within either apoptotic smooth muscle cells or macrophages located in the fibrous cap (44), both features of higher risk plaques, confirming a link between biological processes leading to plaque rupture and PSS.

While these studies demonstrate that high PSS is associated with multiple features of higher-risk plaques, high PSS is also associated with morphological evidence of rupture. For example, post-mortem studies demonstrate that high PSS regions correlate with intimal tears, and site of tearing is influenced by variations in the mechanical strength of cap tissue (45), suggesting that the combination of high PSS and focal weak points lead to plaque rupture.

## Clinical Studies Assessing Pss And Plaque Rupture

High PSS is also associated with features of higher risk plaques in *in vivo* clinical imaging studies, which also demonstrate that PSS provides incremental prognostic information to imaging. For example, *in vivo* IVUS-based assessment in a 3D model demonstrated that coronary PSS increases with increasing lipid core, but reduces with decreasing luminal area and increasing calcification (42). Furthermore, FEA applied to several VH-IVUS clinical studies (Table 1) showed that patients with ACS presentations had higher PSS in high-risk plaque regions such as PB>70%, MLA ≤ 4 mm^2^, and in VH-TCFA than stable angina patients, and inclusion of PSS significantly improved ability of these high-risk features to predict ACS (15). Similarly, PSS increased with increasing lumen area, lumen eccentricity and necrotic core in fibroatheromas, and PSS was higher in OCT-defined ruptured plaques compared with stable lesions (46).

**Table 1:** Recent clinical studies assessing the effect of PSS in coronary atherosclerosis.

**Reference**	**Sample size (n)**	**Imaging used for computational simulation**	**Outcome description**
Teng et al. (15)	53	VH-IVUS	•↑PSS in non-calcified VH-TCFA vs. VH-ThCFA • ↑PSS in patients with ACS, where mean luminal area ≤ 4mm^2^, and PB≥70% • PSS increased the positive predictive value for VH-IVUS to identify clinical presentation
Brown et al. (16)	170	VH-IVUS	•↑PSS in MACE lesions at higher-risk regions, including PB≥70% and TCFA • PSS improved the ability of VH-IVUS to predict MACE in plaques with PB≥70% and MLA ≤ 4mm^2^ • Plaques responsible for MACE had larger superficial calcium inclusions that acted to increase PSS
Costopoulos et al. (46)	64	VH-IVUS	•Ruptured FAs had ↑PSS and ↑variation in PSS than non-ruptured FAs • ↑PSS in proximal segments to the rupture sites compared to distal
Costopoulos et al. (47)	40	Angiography for CFD, and VH-IVUS for FEA	•In plaque progression: ↑PSS was associated with larger ↑NC and small ↑FT • In plaque regression: ↑PSS was associated with ↑NC and ↓FT • ↓WSS was associated with ↑PB • PSS and WSS were independent of each other
Costopoulos et al. (17)	101	VH-IVUS	•↑PSS in the MLA regions of non-culprit MACE lesions • ↑PSS heterogeneity index (HI) in non-culprit MACE than in no-MACE VH-TCFAs • Inclusion of PSS improved the identification of non-culprit MACE lesions • Incorporation of HI further improved the ability of PSS to identify MACE non-culprit lesions
Gu et al. (48)	60	Serial VH-IVUS	•The relationship between ΔPSS and PB differed between high-intensity statin (HIS) and control groups • ↑PSS in control lesions with PB>60% but not with HIS treatment • ΔPSS correlated with changes in lumen curvature, irregularity and roughness, all of which were ↓ in HIS
Doradla et al. (50)	30	IVUS and OCT	•A multifactorial stress equation (MSE) is derived to calculate the peak stress matric, which showed excellent correlation with FEA-derived peak stress • In coronary segments with plaque ruptures, the MSE located the rupture site
Huang et al. (24)	37	OCT	•Maximal ΔPSS gradient was observed at the proximal shoulder, and intermediate at minimal lumen area • Larger relative lumen deformation and ΔPSS were observed in diseased segments compared with normal segments • ΔPSS was positively correlated with plaque burden and negatively correlated with fibrous cap thickness

*ACS, acute coronary syndrome; CFD, computational fluid dynamics; FA, fibroatheroma; FEA, finite element analysis; FT, fibrous tissue; IVUS, intravascular ultrasound; MACE, major adverse cardiovascular events; MLA, minimum lumen area; NC, necrotic core; OCT, optical coherence tomography; PB, plaque burden; PSS, plaque structural stress; TCFA, thin-cap fibroatheroma; ThCFA, thick-cap fibroatheroma; VH, virtual histology; WSS, wall shear stress*.

The ability of PSS to predict future MACE in higher-risk non-culprit lesions has been examined by 2 VH-IVUS studies. Baseline PSS was increased in 22 plaques leading to MACE vs. 22 propensity-matched control lesions in patients from the 170-patient VH-IVUS in vulnerable atherosclerosis (VIVA) study, and improved the ability of imaging to predict events (16). Similarly PSS was increased in 35 non-culprit MACE plaques vs. 66 propensity-matched lesions in patients from the Providing Regional Observations to Study Predictors of Events in the Coronary Tree (PROSPECT) study, and high PSS and longitudinal heterogeneity of PSS were both associated with future MACE (17). High PSS has also been associated with site of rupture *in vivo*, and the association between high PSS and plaque rupture is also not confined to coronary arteries. For example, ruptured coronary plaques on IVUS show higher PSS than matched unruptured plaques (46), and carotid plaques with prior ruptures have higher PSS compared with non-ruptured plaques on magnetic resonance imaging (34), while high PSS on pre-rupture computed tomography is seen in carotid plaques that subsequently ruptured (35).

These studies show how PSS is affected by plaque composition and increased in higher-risk lesions. However, PSS can also be combined with other biomechanical analyses to examine the relationship between different plaque stresses on plaque progression and rupture. For example, combining ESS and PSS showed that baseline ESS and PSS were largely independent of each other irrespective of PB (47). Lower baseline ESS was associated with increased plaque burden over time, while higher baseline PSS was associated with a greater increase in necrotic core, coinciding with higher ESS. The largest increase in fibrous tissue occurred with low ESS and high PSS, and vice-versa, demonstrating that low ESS mostly affects plaque progression, while high PSS mostly affects development of a higher-risk plaque phenotype (47). PSS is also affected by drug treatment over time. For example, serial VH-IVUS imaging showed that changes in PSS over time are dependent on baseline disease severity and medical treatment, mediated in part through remodeling artery geometry and plaque microstructure (48).

## Impact Of Identifying Plaque Stability And Pss Calculations On Clinical Practice

Plaque imaging aims to identify plaques at higher risk of MACE, so that drug or interventional therapy can be adjusted. Incremental prognostic information gained from PSS calculations could therefore be used to refine risk-prediction, and thus improve targeting of treatment to low- and high-risk patients. However, the value of either preventive stenting or aggressive lipid lowering based on finding higher-risk non-culprit lesions is currently unproven, although the subject of a number of research studies. For example, stenting of non-flow-limiting vulnerable plaques based on OCT and NIRS appearances was associated with favorable outcome of treated vessels in the PROSPECT-ABSORB trial. While promising, this trial did not demonstrate that MACE were reduced in the stented patients, and this requires proof from other trials.

## Limitations Of Pss Modeling

While biomechanical simulation of PSS shows promise for both understanding relationships between plaque geometry, architecture and composition with risk of rupture and events, the current techniques have limitations. First, materials are assumed to be isotropic and incompressible, but atherosclerotic plaques are not isotropic, and differences in radial and circumferential moduli are not included in current methods. Second, homogeneous material properties are assumed, and spatial and inter-patient variations within a particular component are not considered. Third, maximum plaque stresses do not necessarily correspond to regions of actual rupture, which may occur at the second or third highest stress region, possibly because *in vivo* materials have more complex characteristics including weaker fibrous caps at these regions. Fourth, intravascular imaging modalities used to provide patient-specific plaque geometry for biomechanical modeling have varying degrees of accuracy, such as the inability of VH-IVUS to measure FCT and OCT penetration to detect depth. Furthermore, the inflammatory state of the atherosclerotic plaque, reflected by features such as macrophage infiltration or pro-inflammatory cytokine expression, may be important in determining plaque material properties, and no reliable methods currently exist to incorporate inflammation into finite element models. In addition, while biomechanical modeling can be applied to both stable and unstable plaques, careful reconstruction of pre-ruptured plaque structures is required since vessel structure is altered after rupture. Finally, computational modeling requires trained experts to process medical images and run simulations that require high computational time and power, and thus its use in clinical settings to provide real-time analysis is underdeveloped.

## Future Perspectives

Future clinical utility of *in vivo* PSS calculation from real-time imaging relies on continuing advances in medical imaging and computational methods. Hybrid dual-probe IVUS-OCT catheters have been developed to overcome limitations of current intravascular imaging techniques, allowing high-resolution plaque surface imaging with large penetration depth (25). Artificial intelligence has been used to automate plaque characterization (23, 49) to bring real-time computer simulation one step closer to reality. New methods to model biomechanical profiles of human coronary plaques using either combined IVUS and OCT or OCT alone for plaque stress simulation have also been described (24, 50). These methods show potential but are based on small studies (summarized in Table 1), and further studies are required to assess their usefulness in prognostic evaluation of atherosclerotic plaques.

## Conclusion

Identification of vulnerable or higher-risk plaques has rested predominantly on pathological studies, more recently supplemented by *in vivo* coronary imaging to detect analogous features. In contrast, biomechanical determinants of propensity to rupture are less studied, in part because of the imaging modalities required and complexity and assumptions made for biomechanical modeling. However, computer simulations have allowed significant advances in biomechanical analysis of atherosclerosis, and modeling arteries as simple cylinders provides at least a conceptual insight, if not a precise quantification. Despite these limitations, advances in imaging and computational methods improve our understanding of these biomechanical processes, and could achieve better cardiovascular risk stratification and management.

## Author Contributions

SG and MB conceived the idea and wrote the first draft. Both authors contributed substantially to the discussion of content and reviewed/edited the manuscript before submission.

## Funding

This work was funded by British Heart Foundation (BHF) grants FS/19/66/34658, PG/16/24/32090, RG71070, RG84554, the National Institute of Health Research Cambridge Biomedical Research Center, and the BHF Center for Research Excellence.

## Conflict of Interest

The authors declare that the research was conducted in the absence of any commercial or financial relationships that could be construed as a potential conflict of interest.

## Publisher's Note

All claims expressed in this article are solely those of the authors and do not necessarily represent those of their affiliated organizations, or those of the publisher, the editors and the reviewers. Any product that may be evaluated in this article, or claim that may be made by its manufacturer, is not guaranteed or endorsed by the publisher.
